# Cervical lymph node metastasis prediction of postoperative papillary thyroid carcinoma before ^131^I therapy based on clinical and ultrasound characteristics

**DOI:** 10.3389/fendo.2023.1122517

**Published:** 2023-02-17

**Authors:** Fei Yu, Wenyu Wu, Liuting Zhang, Shaohua Li, Xiaochen Yao, Jun Wang, Yudan Ni, Qingle Meng, Rui Yang, Feng Wang, Liang Shi

**Affiliations:** ^1^ Department of Nuclear Medicine, Nanjing First Hospital, Nanjing Medical University, Nanjing, China; ^2^ Department of Functional Examination, Nanjing First Hospital, Nanjing Medical University, Nanjing, China

**Keywords:** papillary thyroid carcinoma, lymph node metastasis, nomogram, ^131^I, Tg, TgAb, ultrasound

## Abstract

**Background:**

The status of lymph nodes is crucial to determine the dose of radioiodine-131(^131^I) for postoperative papillary thyroid carcinoma (PTC). We aimed to develop a nomogram for predicting residual and recurrent cervical lymph node metastasis (CLNM) in postoperative PTC before ^131^I therapy.

**Method:**

Data from 612 postoperative PTC patients who underwent ^131^I therapy from May 2019 to December 2020 were retrospectively analyzed. Clinical and ultrasound features were collected. Univariate and multivariate logistic regression analyses were performed to determine the risk factors of CLNM. Receiver operating characteristic (ROC) analysis was used to weigh the discrimination of prediction models. To generate nomograms, models with high area under the curves (AUC) were selected. Bootstrap internal validation, calibration curves and decision curves were used to assess the prediction model’s discrimination, calibration, and clinical usefulness.

**Results:**

A total of 18.79% (115/612) of postoperative PTC patients had CLNM. Univariate logistic regression analysis found serum thyroglobulin (Tg), serum thyroglobulin antibodies (TgAb), overall ultrasound diagnosis and seven ultrasound features (aspect transverse ratio, cystic change, microcalcification, mass hyperecho, echogenicity, lymphatic hilum structure and vascularity) were significantly associated with CLNM. Multivariate analysis revealed higher Tg, higher TgAb, positive overall ultrasound and ultrasound features such as aspect transverse ratio ≥ 2, microcalcification, heterogeneous echogenicity, absence of lymphatic hilum structure and abundant vascularity were independent risk factors for CLNM. ROC analysis showed the use of Tg and TgAb combined with ultrasound (AUC = 0.903 for “Tg+TgAb+Overall ultrasound” model, AUC = 0.921 for “Tg+TgAb+Seven ultrasound features” model) was superior to any single variant. Nomograms constructed for the above two models were validated internally and the C-index were 0.899 and 0.914, respectively. Calibration curves showed satisfied discrimination and calibration of the two nomograms. DCA also proved that the two nomograms were clinically useful.

**Conclusion:**

Through the two accurate and easy-to-use nomograms, the possibility of CLNM can be objectively quantified before ^131^I therapy. Clinicians can use the nomograms to evaluate the status of lymph nodes in postoperative PTC patients and consider a higher dose of ^131^I for those with high scores.

## Introduction

The incidence of thyroid cancer has a rapid increase worldwide, and differentiated thyroid cancer (DTC) accounts for more than 90% of thyroid cancer cases (1–3). Papillary thyroid carcinoma (PTC) is the most common type of DTC and cervical lymph node metastasis (CLNM) frequently occurs in PTC (4, 5). Previous researches identified the rate of CLNM reaches approximately 20-30% DTC after systemic treatment (4, 6). CLNM can be classified as central and lateral lymph node metastasis. CLNM was an important risk factor for recurrence in PTC (7, 8). We admit that, although with controversy, lateral lymph node metastasis but not central lymph node metastasis affected the recurrence-free survival as some recent studies reported (9–11).

Ultrasonography has been widely applied to detect CLNM in PTC but displays a relatively low sensitivity (12, 13). Precisely identifying CLNM plays a vital role in the determination of whether central lymph node dissection and/or lateral lymph node dissection should be performed during surgery. To date, some prediction models have been established for preoperative prediction of CLNM in PTC patients. It was discovered that clinical characteristics including age, gender, tumor size, extrathyroidal invasion, multifocality, serum thyroglobulin (Tg), serum thyroglobulin antibodies (TgAb), radiomic and ultrasound characteristics might predict CLNM in PTC (12, 14–17).

Prophylactic central lymph node dissection is frequently performed in PTC patients with moderate and high-risk, while prophylactic lateral lymph node dissection is not generally recommended as a standard treatment (18). In practice, a significant number of lateral lymph node metastasis can remain after surgery and present as recurrence (19). Residual CLNM after initial surgery are mainly due to overlook of lymph node involvement before surgery or during thyroidectomy, or incomplete removal of involved lymph nodes in surgery (20). Radioiodine-131 (^131^I) therapy that can successfully ablate thyroid cancer cells in both primary and metastatic lesions has been accepted as conventional management for treating moderate and high-risk DTC after operation (18, 21). Radioactive Iodine Ablation reduces the risk of recurrence or death from thyroid cancer patients. The recommended dose for patients receiving it as remnant ablative therapy was 100 mCi. Nonetheless, the ^131^I dose generally needs to be increased to 100-150 mCi and 150-200 mCi when treating patients with CLNM and/or extra-thyroid extension and distant metastasis, respectively (22). Nevertheless, few models were constructed for predicting residual and recurrent CLNM in postoperative PTC patients before ^131^I therapy. Effective prediction of the residual and recurrent CLNM status may provide a reference for nuclear medicine doctors to give appropriate and sufficient ^131^I doses that have enough tumoricidal effects but without increased risk of adverse effects.

Therefore, we aimed to filter out important predictors from medical information and to develop a brief and reliable prediction model (nomogram) for accurate residual and recurrent CLNM in postoperative PTC patients before ^131^I therapy.

## Materials and methods

### Patients

**Figure 1:**
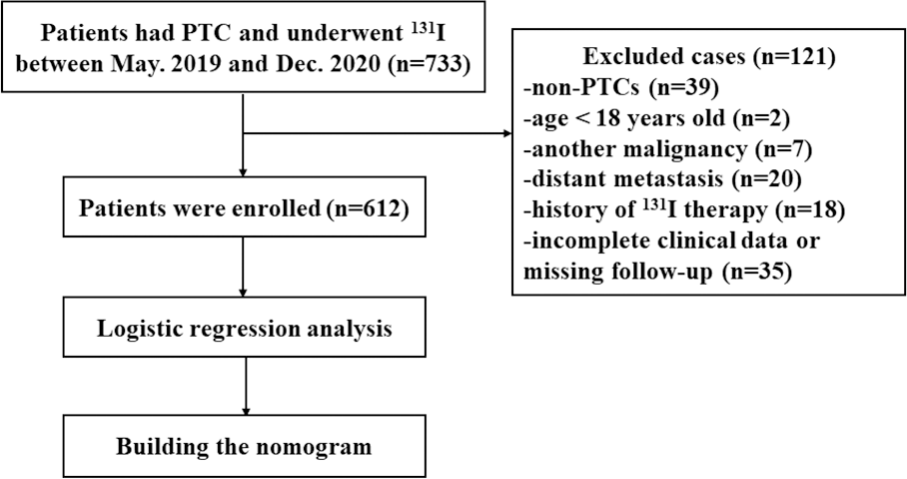
Flow chart of the study selection.

The study was approved by the Ethics Committee of Nanjing First Hospital. Consecutive moderate and high-risk PTC patients who underwent ^131^I therapy from May 2019 to December 2020 at the Department of Nuclear Medicine of Nanjing First Hospital were enrolled. The records of patients were retrospectively reviewed and the exclusion criteria were as follows (1): non-PTCs or other subtypes than classic PTC (such as mixed PTC and so on) (2); age younger than 18 years old (3); patients who underwent non-curable surgery (4); patients with another malignancy (5); patients with distant metastasis (6); history of ^131^I therapy (7); history of neck radiation (8); incomplete clinical data or missing follow-up. According to the above criteria, 612 patients with PTC were enrolled in this study. All patients underwent total/near-total thyroidectomy and central lymph node dissection with/without lateral lymph node dissection. The flow chart of the selection process was shown in **Figure 1**.

### Data collection

Clinicopathological factors included age, gender, histological type, stage, tumor size, multifocal or not, serum Tg level, serum TgAb level, results of cervical ultrasound exams, history of ^131^I therapy and neck radiation were extracted from electronic medical records in our hospital. Multifocality was defined as two or more tumors in the thyroid. The time interval for the first ^131^I therapy was 3 to 6 months after surgery. Serum Tg level, serum TgAb level and results of ultrasound were measured within one week before ^131^I therapy in our hospital. Metastasis status of lymph node evaluated by ultrasound was recorded as yes or no. According to overall ultrasound results, one suspicious (when overall ultrasound reported suspicious metastasis) or one normal lymph node (when overall ultrasound reported negative metastasis) was selected to collect ultrasound features including aspect transverse ratio (≥ 2/< 2), cystic change (yes/no), microcalcification (yes/no), mass hyperecho (yes/no), echogenicity (heterogeneous *vs.* homogeneous), lymphatic hilum structure (presence *vs.* absence) and vascularity (abundant *vs.* non-abundant). The ultrasound features were read and recorded by two experienced ultrasound doctors. The golden standard of metastasis status of cervical lymph nodes before ^131^I was based on cervical SPECT/CT imaging after ^131^I treatment and follow-up (follow-up time was 12 months). In this study, any residual activity noted on ^131^I SPECT/CT scan, pathology-proved CLNM during follow-up and imaging revealed suspicious lymph nodes with continuous abnormal Tg during follow-up were defined as CLNM.

### Statistical analyses

All statistical analyses were performed using R software version 4.2.1. Continuous variables with normal distribution were expressed as the means ± standard deviations (SD), continuous variables with abnormal distribution were expressed as the median (interquartile range), and categorical variables were reported as numbers and percentages.

A t-test, log-rank test, Pearson’s chi-square test or Fisher’s exact test was used to compare the baseline characteristics of different groups. Univariate and multivariate backward stepwise logistic regression analyses were used to assess risk factors for CLNM in postoperative PTC patients. Receiver operating characteristic (ROC) curves were used to test the discrimination of our established prediction models and area under the curves (AUC) were compared by Delong test. For models with high AUC values, nomograms were developed to calculate the probability of CLNM. The performance of the nomogram was further evaluated by the calibration chart, which plotted the predicted probability of the nomogram against the observed probability. Bootstrap method (1,000-times) was used for internal validation of the model. A decision curve analysis was also performed to test the clinical usefulness of the nomogram. All tests are two-sided tests and statistical significance level was established at *P* < 0.05.

## Results

### Patients and clinical characteristics

Baseline characteristics of patients are summarized in **Table 1**. Among the 612 postoperative PTC patients, including 225 men and 387 women, the average age was 42.44 ± 12.23 years old (ranging from 18 to 79 years old). CLNM was found in 115(18.79%) patients. There were no significant differences in terms of patient age, gender, stage, tumor size and multifocality in the postoperative PTC patients with and without CLNM. The positive rate of ultrasound diagnosis was significantly higher in patients with CLNM (73.91%) than those without CLNM (2.01%). Patients with CLNM had a median Tg level of 8.98 ug/l (interquartile range: 0.89-39.60), while patients without CLNM had a median Tg level of 3.00 ug/l (interquartile range: 0.48-10.23) (*P* < 0.001). Additionally, we found a marginally significant (*P* = 0.081) difference of TgAb (IU/ml) between the two groups (with CLNM: median 2.02, interquartile range 1.02-24.20; without CLNM: median 1.71, interquartile range 0.98-7.15).

**Table 1:** Demographics and clinical characteristics of patients.

Variables	PTC with CLNM (n=115)	PTC without CLNM (n=497)	*P*
Age (years)			
Mean ± SD	42.55 ± 11.98	42.42 ± 12.29	0.918
Gender [n (%)]			
male	40 (34.78)	185 (37.22)	
female	75 (65.21)	312 (62.78)	0.703
Stage [n (%)]			
I	106 (92.17)	437 (87.93)	
II	9 (7.82)	45 (9.05)	
III	0 (0)	10 (2.01)	
IV	0 (0)	5 (1.01)	0.395
Tumor size (cm)			
median (interquartile range)	1.30 (0.90-2.00)	1.40 (1.00-2.00)	0.704
Multifocality [n (%)]			
No	64 (55.65)	277 (55.73)	
Yes	61 (44.34)	220 (44.27)	1.000
Ultrasound [n (%)]			
negative	30 (26.09)	487 (97.99)	
positive	85 (73.91)	10 (2.01)	< 0.001
Tg (ug/l)			
median (interquartile range)	8.98 (0.89-39.60)	3.00 (0.48-10.23)	< 0.001
TgAb (IU/ml)			
median (interquartile range)	2.02 (1.02-24.20)	1.71 (0.98-7.15)	0.081

CLNM, cervical lymph node metastasis; SD, Standard deviation; Tg, Thyroglobulin; TgAb, thyroglobulin antibodies.

### Univariate and multivariate predictors for CLNM

The present study identified the potential risk factors for CLNM using univariate and multivariate logistic regression analysis. As shown in **Table 2**, The univariate analysis identified that levels of Tg (*P* < 0.001), levels of TgAb (*P* = 0.009), overall ultrasound (*P* < 0.001) and seven ultrasound features (all *P* < 0.001) were statistically significant associated with CLNM. CLNM was not significantly related to age, gender, stage, tumor size and multifocal (*P* > 0.05). In the multivariate backward stepwise analysis, Tg (*P* =2.290×10^-3^), TgAb (*P* =6.910×10^-3^) and overall ultrasound (*P* < 0.001) were significantly associated with CLNM. When considering seven ultrasound features, Tg, TgAb and all seven ultrasound features were finally retained in the multivariate model. However, TgAb, cystic change, and mass hyperecho didn’t reach statistical significance (P =0.147, 0.060 and 0.091, respectively). These results revealed that levels of Tg, levels of TgAb, and ultrasound may be independent risk factors associated with CLNM in PTC patients before ^131^I therapy.

**Table 2:** Univariate and multivariate stepwise logistic regression analysis of risk factors for CLNM in postoperative PTC before ^131^I therapy.

Variables	Univariate analysis		Multivariate analysis ^a^		Multivariate analysis ^b^	
	OR (95% CI)	*P*	OR (95% CI)	*P*	OR (95% CI)	*P*
**Age** (>40 *vs.* ≤40 years) ^*^	1.08 (0.72-1.62)	0.707	NA		NA	
**Gender** (female *vs.* male)	1.11 (0.73-1.71)	0.625	NA		NA	
**Stage** (II-IV *vs.* I)	0.62 (0.28-1.23)	0.198	NA		NA	
**Tumor size** (>1.4 cm *vs.* ≤1.4 cm) ^*^	0.92 (0.61-1.38)	0.689	NA		NA	
**Multifocality** (yes *vs.* no)	1.00 (0.67-1.51)	0.987	NA		NA	
**Tg** (>12.58 *vs.* ≤12.58 ug/l) ^#^	3.16 (2.06-4.86)	<0.001	3.23 (1.52-6.94)	2.290×10^-3^	3.13 (1.53-6.45)	1.797×10^-3^
**TgAb** (>13.20 *vs.* ≤13.20 IU/ml) ^#^	1.83 (1.16-2.86)	8.580×10^-3^	2.96 (1.34-6.54)	6.910×10^-3^	1.75 (0.81-3.70)	0.147
**Overall ultrasound** (positive *vs.* negative)	137.98 (67.90-308.87)	<0.001	125.31 (60.49-286.61)	<0.001	NA	
**Ultrasound features:**			NA			
**Aspect transverse ratio** (≥2 *vs.* <2)	10.34 (6.60-16.49)	<0.001			3.69 (1.96-6.92)	<0.001
**Cystic change** (yes *vs.* no)	63.22 (12.39-1154.83)	<0.001			8.54 (1.28-172.28)	0.060
**Microcalcification** (yes *vs.* no)	27.49 (14.78-54.25)	<0.001			3.99 (1.52-11.11)	6.217×10^-3^
**Mass hyperecho** (yes *vs.* no)	34.31 (9.40-220.66)	<0.001			4.55 (0.92-35.17)	0.091
**Echogenicity** (heterogeneous *vs.* homogeneous)	37.40 (22.11-65.20)	<0.001			6.46 (2.69-15.24)	<0.001
**Lymphatic hilum structure** (absence *vs.* presence)	11.63 (6.01-23.54)	<0.001			5.64 (2.16-14.98)	<0.001
**Vascularity** (abundant *vs.* non-abundant)	27.59 (11.88-75.42)	<0.001			4.00 (1.28-14.08)	0.022

^*^ Patients were grouped by median of the variable from all patients, ^#^ Patients were grouped by cut-off value of receiver operating characteristic curve analysis, ^a^ Multivariate stepwise logistic regression analysis of overall ultrasound and other variables, ^b^ Multivariate stepwise logistic regression analysis of ultrasound features and other variables.

CLNM, cervical lymph node metastasis; PTC, papillary thyroid carcinoma; Tg, Thyroglobulin; TgAb, thyroglobulin antibodies; OR, Odd ratio; CI, Confidence intervals; NA, Unavailable.

### ROC analysis of different models

**Figure 2:**
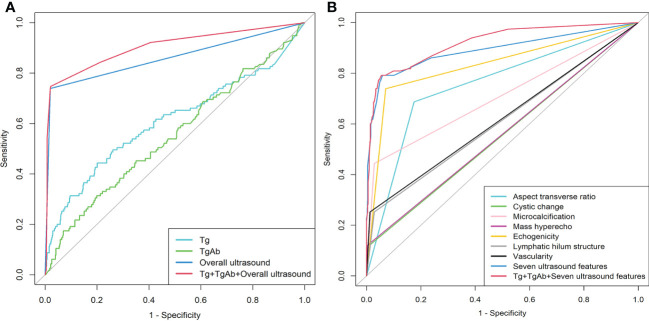
Receiver operating characteristic curve analysis of different prediction models for predicting CLNM in postoperative PTC before ^131^I therapy. **(A)** ROC curves for Tg, TgAb, overall ultrasound and combined models. **(B)** ROC curves for seven ultrasound features and combined models.

**Figure 2** illustrates the ROC curves of the CLNM prediction results of the thirteen models. According to the results, the AUCs of the models achieved 0.608 (Tg), 0.552 (TgAb), 0.860 (overall ultrasound), 0.756 (aspect transverse ratio), 0.556 (cystic change), 0.708 (microcalcification), 0.559 (mass hyperecho), 0.834 (echogenicity), 0.612 (lymphatic hilum structure), 0.620 (vascularity), 0.892 (seven ultrasound features), 0.903 (Tg+TgAb+Overall ultrasound), and 0.921 (Tg+TgAb+seven ultrasound features), respectively. The corresponding quantitative indexes of all the above models were summarized in **Table 3**. Based on Delong test, seven ultrasounds aspects combined model performed better than the overall ultrasound model (*P* = 0.0321), “Tg+TgAb+Overall ultrasound” model performed better than Tg, TgAb, and Overall ultrasound models (all *P* < 0.01), “Tg+TgAb+Seven ultrasound features” model performed better than Tg, TgAb, each ultrasound feature and seven ultrasound features combined models (all *P* < 0.01). Nevertheless, we didn’t observe a better performance of “Tg+TgAb+Seven ultrasound features” model than “Tg+TgAb+Overall ultrasound” model (*P* = 0.248).

**Table 3:** Quantitative indexes of different models.

Models	AUC	Cut-off	Sensitivity	Specificity
Tg	0.608 (0.543-0.674)	12.58	0.443	0.799
TgAb	0.552 (0.491-0.614)	13.20	0.313	0.801
Overall ultrasound	0.860 (0.819-0.900)	/	0.739	0.980
Aspect transverse ratio	0.756 (0.710-0.802)	/	0.687	0.825
Cystic change	0.556 (0.526-0.585)	/	0.113	0.998
Microcalcification	0.708 (0.662-0.754)	/	0.443	0.972
Mass hyperecho	0.559 (0.529-0.589)	/	0.122	0.996
Echogenicity	0.834 (0.793-0.876)	/	0.739	0.93
Lymphatic hilum structure	0.612 (0.572-0.653)	/	0.252	0.972
Vascularity	0.620 (0.580-0.660)	/	0.252	0.988
Seven ultrasound features	0.892 (0.853-0.932)	/	0.791	0.942
Tg+TgAb+Overall ultrasound	0.903 (0.866-0.940)	/	0.748	0.978
Tg+TgAb+Seven ultrasound features	0.921 (0.890-0.951)	/	0.791	0.946

AUC, area under curve; Tg, Thyroglobulin; TgAb, thyroglobulin antibodies.

### Construct a nomogram for predicting risk of CLNM

**Figure 3:**
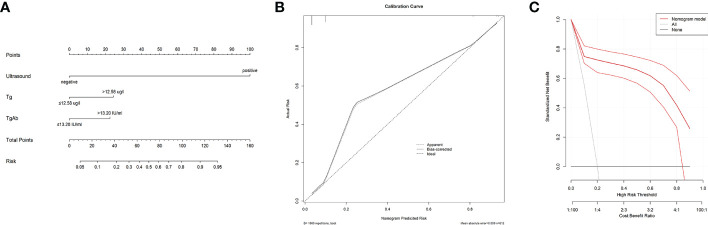
The nomogram, calibration curve and decision analysis curve of “Tg+TgAb+Overall ultrasound” model for predicting CLNM in postoperative PTC before ^131^I therapy. **(A)** A nomogram incorporating serum Tg, serum TgAb and overall ultrasound was constructed. **(B)**The calibration curve of the nomogram model. The x-axis showed the predictive risk by nomogram, and the y-axis represented the actual CLNM risk. The ideal and bias-corrected risk were presented with the dotted black and solid black lines, respectively. **(C)** Decision curve analysis of the model. The x-axis showed threshold probability and the y-axis represented net benefit. The solid red line displayed the benefit and 95% confidence interval of the developed nomogram.

**Figure 4:**
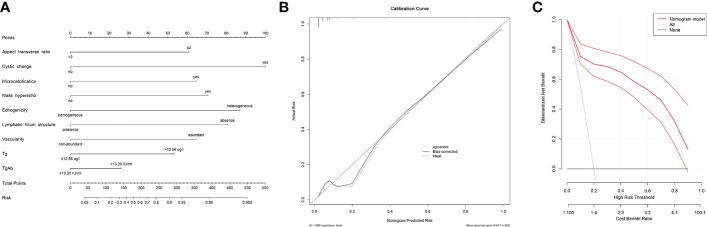
The nomogram, calibration curve and decision analysis curve of “Tg+TgAb+ Seven ultrasound features” model for predicting CLNM in postoperative PTC before ^131^I therapy. **(A)** A nomogram incorporating serum Tg, serum TgAb and seven ultrasound features was constructed. **(B)** The calibration curve of the nomogram model. The x-axis showed the predictive risk by nomogram, and the y-axis represented the actual CLNM risk. The ideal and bias-corrected risk were presented with the dotted black and solid black lines, respectively. **(C)** Decision curve analysis of the model. The x-axis showed threshold probability and the y-axis represented net benefit. The solid red line displayed the benefit and 95% confidence interval of the developed nomogram.

We chose two models which showed good discrimination (AUC exceeding 0.9) to construct nomograms. As shown in **Figure 3A**, the nomogram was constructed and incorporated variables including Tg, TgAb, and overall ultrasound. Nomogram in **Figure 4A** contained Tg, TgAb, and seven ultrasound features. For individualized prediction, draw an upward vertical line from the patient’s characteristics to calculate total points. Then, the risk of CLNM in each patient can be determined based on the total points. The two nomograms were cross-validated internally by the 1000 repetitions of bootstrap samples and the C-index were 0.899 and 0.914, respectively. **Figures 3B**, **4B** showed the calibration curves of “Tg+TgAb+Overall ultrasound” model and “Tg+TgAb+Seven ultrasound features” model. The unreliability test yielded a *P* value of 0.966 and 0.927, respectively, indicating a good concordance between the predicted and actual outcomes. Ultimately, we used decision analysis curves to estimate the clinical utility of our nomograms, and results showed that the two nomograms had satisfactory net benefits among most of the threshold probabilities (**Figures 3C**, **4C**).

## Discussion

Due to unrecognized occult lymph nodes preoperatively or incomplete removal of metastatic lymph nodes during surgery, residual CLNM after initial surgical treatment for PTC is common (20). Radioactive iodine ablation is highly effective for treating CLNM in postoperative PTC patients (23). As thyroid cancer cells can’t uptake the radioactive iodine as easily as normal thyroid cells, higher administered activities (up to 150 mCi) are generally recommended for PTC patients with remnant CLNM to achieve a good therapeutic effect (18). Therefore, accurate identification of CLNM can help provide personalized ^131^I doses and then improve the prognosis of PTC patients. In the current study, 115 (18.79%) PTC patients presented residual and recurrent CLNM before taking ^131^I. We identified independent risk factors associated with CLNM as follow: higher Tg, higher TgAb, positive overall ultrasound and ultrasound features such as aspect transverse ratio ≥ 2, microcalcification, heterogeneous echogenicity, absence of lymphatic hilum structure and abundant vascularity.

Our study found serum Tg levels higher than 12.58 ug/l and TgAb higher than 13.20 IU/ml were related to higher CLNM risk in postoperative PTC patients. Serum Tg and TgAb are two important biomarkers that serial measurements of them are beneficial to the postoperative management of DTC patients (24, 25). Previous researches also proved the role of Tg and TgAb in predicting CLNM in DTC patients before and after surgery. Zou et al. found that ipsilateral lateral CLNM was more likely to occur before surgery when Tg > 100.01 ug/l or TgAb > 89.43 IU/ml (26). Tg and TgAb detection from fine-needle aspiration of cervical lymph node can determine metastasis with a cut-off value of 227.1 ug/l and 10.85 IU/ml, respectively (27). On the other hand, Zhang et al. identified serum Tg but not TgAb can detect recurrence and metastasis of DTC after surgery and ^131^I treatment with an accuracy of 77.5% (28). In DTC patients complicated by Hashimoto thyroiditis after surgery and ^131^I treatment, Chai et al. revealed the optimal cut-off value associated with metastasis in serum Tg and TgAb was 1.48 µg/L (AUC = 0.907) and 45 IU/ml (AUC = 0.650), respectively (29). Recent studies have demonstrated that PTC might lead to an autoimmune thyroid inflammation characterized by TgAb and anti-thyroperoxidase (TPOAb) (30, 31). In theory, production of TgAb can also be triggered by the presence of Tg (32). Elevated serum TgAb indicated that the tumor is active (33) and our study suggested that TgAb can be an independent predictor for residual and recurrent CLNM in PTC patients. Positive TgAb can interfere with Tg measurement and reduce the reliability of Tg level (34). The inclusion of both Tg and TgAb in our model reduced the risk of underestimating CLNM due to measurement interference to some extent.

In the present study, it was also discovered that general ultrasound and specific ultrasound characteristics, including aspect transverse ratio, microcalcification, echogenicity, lymphatic hilum structure, and vascularity, are independent risk factors for CLNM in postoperative PTC patients. Past research works demonstrated that the sensitivity, specificity and accuracy of ultrasonic diagnosis of CLNM after treatment of DTC were 78.5%, 60.0%, and 75.0%, respectively (28). Our study showed a comparable sensitivity (0.739) and a higher specificity (0.980) in overall ultrasound. Former researchers focused on ultrasound characteristics of thyroid nodules related to CLNM preoperatively and found microcalcifications, abundant blood flow, and an irregular shape were significant risk factors (12, 14, 35, 36). Ultrasound parameters including cystic change and mass hyperecho slightly missed the margin of significance and showed poor discrimination values of AUC (<0.6) in this investigation. Our results indicated that ultrasound doctors should pay more attention to aspect transverse ratio, microcalcification, echogenicity, lymphatic hilum structure, and vascularity when judging CLNM.

The nomogram, a simple and useful tool, was widely used for predicting individual probabilities of clinical events (37, 38). Recently, effective nomograms have been constructed to indicate the risk of lymph node metastasis in different types of cancers (39–42). Similar work has been done before to establish a nomogram for preoperatively predicting central lymph node metastasis in PTC patients (15, 43, 44). Nevertheless, they focused on preoperative prediction of CLNM and ignored the importance of predicting residual and recurrent CLNM risk before ^131^I therapy. Moreover, some studies established the nomogram depends on clinical factors (15, 44) and the accuracy of the model is not high enough (C-index slightly above 0.7). Min et al. collected comprehensive variables like clinical factors, thyroid function tests, and ultrasound features and consequently established a novel nomogram with a favorable C-index of 0.815 (43). To fill the gap that there is no established nomogram for predicting postoperatively residual and recurrent CLNM risk before ^131^I therapy, we collected multiple variates containing clinical, ultrasonic, and hematological data and incorporated risk factors into two easy-to-use nomograms: one based on overall ultrasound and another based on ultrasound features. The two combined models (C-index 0.903 for “Tg+TgAb+Overall ultrasound” model, C-index 0.921 for “Tg+TgAb+seven ultrasound features” model) both showed better diagnostic performance than any single method in differentiating postoperative PTC patients with metastatic and non-metastatic lymph nodes. Internal validation also showed favorable discrimination and calibration. DCA proved that utilizing the two nomograms to predict CLNM before ^131^I therapy would be beneficial at almost any threshold probability. We recommended prior usage of “Tg+TgAb+seven ultrasound features” model to calculate CLNM probability. When ultrasound features cannot be obtained, “Tg+TgAb+overall ultrasound” model is alternative and more convenient to use.

There are some limitations in our research that cannot be ignored. First, this was a retrospective study, which could inevitably cause some selection bias, information bias or confounding bias. Second, several specific data could not be obtained from the medical records, or were missing, including extrathyroidal extension and BRAF mutation status. Third, central lymph node metastasis (N1a) and lateral lymph node metastasis (N1b) showed different prognosis and they will show higher clinical value if we divided CLNM into N1a and N1b. However, the small sample size in N1a prevented us from the stratified analysis. Fourth, this study was conducted in a single institutional center with a limited sample size. In the future, external validation with more cases from multiple centers is needed.

## Conclusion

Based on the clinical information including serum Tg, serum TgAb and ultrasound of postoperative PTC patients, we plotted two nomograms with high accuracy and reliability that could predict residual and recurrent CLNM in PTC patients before ^131^I. We believe that our nomograms can assist clinicians to determine personalized ^131^I dose for PTC patients.

## Data availability statement

The raw data supporting the conclusions of this article will be made available by the authors, without undue reservation.

## Ethics statement

The studies involving human participants were reviewed and approved by the Ethics Committee and Institutional Review Board of Nanjing First Hospital. The patients/participants provided their written informed consent to participate in this study.

## Author contributions

LS and FW contributed to the design of the study. The first draft of the manuscript was written by FY, WW, and LZ. LS contributed to the manuscript review and editing. WW and LZ collect the data. FY performed the data analysis. All authors contributed to the article and approved the submitted version.
